# An updated meta-analysis approach for genetic linkage

**DOI:** 10.1186/1471-2156-6-S1-S43

**Published:** 2005-12-30

**Authors:** Carol J Etzel, Mei Liu, Tracy J Costello

**Affiliations:** 1 The Department of Epidemiology, UT MD Anderson Cancer Center, Houston, TX, USA; 2 The University of Texas School of Public Health, Houston, TX, USA

## Abstract

We present a meta-analysis procedure for genome-wide linkage studies (MAGS). The MAGS procedure combines genome-wide linkage results across studies with possibly distinct marker maps. We applied the MAGS procedure to the simulated data from the Genetic Analysis Workshop 14 in order to investigate power to detect linkage to disease genes and power to detect linkage to disease modifier genes while controlling for type I error. We analyzed all 100 replicates of the four simulated studies for chromosomes 1 (disease gene), 2 (modifier gene), 3 (disease gene), 4 (no disease gene), 5 (disease gene), and 10 (modifier gene) with knowledge of the simulated disease gene locations. We found that the procedure correctly identified the disease loci on chromosomes 1, 3, and 5 and did not erroneously identify a linkage signal on chromosome 4. The MAGS procedure provided little to no evidence of linkage to the disease modifier genes on chromosomes 2 and 10.

## Background

Kofendred Personality Disorder (KPD), as simulated for Genetic Analysis Workshop 14 (GAW14), is a psychiatric syndrome characterized by an overwhelming concern with the meaning of personal inner emotions while regarding the emotions of others. Like other complex personality disorders, KPD has numerous behavioral and biological characteristics. Additionally, KPD, like other complex diseases, is believed to be linked to many genes. The possibility of finding the majority of these genes from one independent study is small. Instead, pooling data across independent studies (i.e., a mega-analysis) or pooling linkage results across independent studies (i.e., a meta-analysis) may be the best means to identify these numerous genes with small effects.

In a mega-analysis, combining raw data from several studies allows the investigator to increase sample size. A mega-analysis can lead to an increase in power to detect linkage and reduce the level of type I error. Combining raw data would be an ideal approach, but data are not always readily available or freely shared. In a meta-analysis, the investigator can still combine information from several studies to obtain a consensus for linkage. The information typically found in the literature can range from published *p*-values, LOD scores, or effect sizes.

Caveats to mega- and meta-analyses involve among-study heterogeneity, which can include differing marker maps, informativeness, sample sizes, sampling plans, and linkage tests. Methods have been proposed to handle such problems. The genome-scan meta-analysis (GSMA) method proposed by Wise et al. [1] accommodates differing marker maps in a meta-analysis, but this test is based on the level of significance (magnitude of LOD score or *p*-value) at each marker. Combining results from significance tests can be limited [2-4] where the concordance or discordance of significant linkage between two studies may not reflect the existence of true linkage, but rather may be based on the amount of heterogeneity between the two studies. Combining effect sizes may be a better approach than combining results from significance tests, but there are still limitations if the studies have differing marker maps and use different tests to evaluate linkage. Etzel and Guerra [5] developed a method to evaluate evidence for linkage to a QTL from several linkage studies. However, this method has not been tested for a genome-wide scan and it requires that all studies use the same type of linkage test (i.e., some version of the Haseman-Elston test). Loesgen et al. [6] developed a meta-analytic method that computes a weighted average estimate of score statistics where one proposed weighting scheme is a function of information content at a marker and sample size. Although this method was first proposed for studies using a common marker map, it can be extended to combine studies with differing marker maps.

In this paper, we present an updated meta-analysis method for assessing linkage to a quantitative trait locus (QTL) that generalizes the meta-analytic procedure first proposed by Etzel and Guerra [5] such that it does not assume that all studies use the same test for linkage and extends the weighting procedure proposed by Loesgen et al. [6] to incorporate differing marker maps. The result from the meta-analysis procedure of genome-wide linkage studies (MAGS) method is a genome-wide weighted average of evidence of linkage to a complex disease. Although this approach was developed to evaluate linkage to a QTL, we applied it to evaluate evidence of linkage to KPD (affection status) using the four simulated data sets provided for the GAW14 with knowledge of the disease gene locations.

## Methods

### The MAGS procedure

The MAGS method that we developed is based on procedures proposed by Loesgen et al. [6] and Etzel and Guerra [5]. For MAGS, it is not assumed that the studies use the same marker map or that they use the same test for linkage. However, it is assumed that the marker maps are available as well as the sample size, information content at each marker (preferred), and linkage summary statistics (LOD scores, nonparametric linkage (NPL) scores, or *p*-values).

The MAGS method is based on a weighted average of transformed normal variates that are obtained through the reported linkage summary statistics. Suppose that we wish to complete a meta-analysis on *k *studies. Each study *k *has *m*_*k *_number of markers. It is not assumed that the studies have the same number of markers, *m*_*i *_≠ *m*_*k*_, *i *≠ *j *nor it is assumed that the studies have the same marker maps. For a specified chromosome, let *M*_*st *_denote the *t*^th ^marker from study *s*, for *s *= 1, ..., *k *and *t *= 1, ..., *m*_*k*_. Define {*L*_*q*_,*q *= 1, ..., *l*} as the set of analysis points such that the *L*_*q *_are equally spaced across the chromosome. For each set of *M*_*st *_on a chromosome,

1. Transform the summary statistic for each marker *M*_*st *_to a *p*-value, *p*_*st*_, for example

a. HLOD to Chi-square variate: *X*_*st *_= 4.6* *HLOD*_*st *_and obtain a *p*-value for each chi-square variate [7]: 

b. Transform NPL to a *p*-value: *p*_*st *_= Pr(*Z *<*NPL*_*st*_). This *Z *is not necessarily a standard normal random variable. Rather, *Z *is a normal variable with mean 0 and standard deviation of *σ*^2^. The calculation of *σ*^2 ^is difficult and may be influenced by incomplete information. However, we have found that in practice (data not shown) that this situation does not erroneously affect the meta-analysis.

2. Transform the resulting *p*-value to a normal variate: *Z*_*st *_= Φ^-1 ^(*P*_*st*_)

3. For each analysis point *L*_*q*_, calculate the weighted normal variate:



where *w*_*stq *_is the weight given to marker *M*_*st *_and *Z*_*st *_is the normal variate for marker *M*_*st*_. The indicator function  is defined as 1 if marker *M*_*st *_is within a set distance *D *cM from analysis point *L*_*q *_and 0 otherwise. The weight *w*_*stq *_for marker *M*_*st *_can be a function of study sample size, information content at that marker, and/or distance (recombination fraction, *θ*_*stq*_) between marker *M*_*st *_and analysis point *L*_*q*_, say .

4. Calculate the *p*-value for each meta-analytic variate: 

The *p*-values from step 4 can then be compared to a set level to determine areas with combined evidence for linkage. NOTE: If all studies use the same marker map, then the combined set of markers can replace the analysis points *L*_*q *_and step 3 simplifies to 

### MAGS applied to GAW 14 simulated microsatellite datasets

The provided genome-wide microsatellite marker maps were identical for all four simulated data sets. We used GENEHUNTER2 [8,9]) to assess evidence for linkage to KPD for all 100 replicates of chromosomes 1, 2, 3, 4, 5, and 10 within all four studies. We then applied the MAGS method as described above to the linkage results, one chromosome at a time. Within each chromosome replicate the NPL scores obtained from GENEHUNTER2 were transformed to *p*-values using step 1b. The subsequent normal variates were weighted by *w*_*st *_= *I*_*st*_*n*_*s*_, where *n*_*s *_is defined as the total number of individuals used in the linkage analysis of study *s *and *I*_*st *_is the information content (given as output from GENEHUNTER2) for marker *t *in study *s*. For AI, *n*_*1 *_= 683, for DA, *n*_*2 *_= 700, for KA *n*_*3 *_= 694, and for study NY, *n*_*4 *_= 943. Since the four studies had identical marker maps, we used the simplified version of step 3 to calculate the weighted MAGS estimate  for each marker location *t*.

### MAGS applied to GAW 14 simulated single-nucleotide polymorphism (SNP) datasets

The provided genome-wide SNP marker maps were also identical for all 4 simulated data sets. In order to fully evaluate the MAGS method using studies with differing marker maps and different analysis packages, we created unique genome-wide SNP marker maps for each study for chromosomes 1, 2, 3, and 10. The unique marker maps were created by simply randomly assigning the available markers on each chromosome to one of the four studies. We then used a different linkage test within each study to assess linkage to KPD one chromosome at a time: the Sibpal procedure from SAGE [10] for study AI, GENEHUNTER2 [8,9] for studies DA and KA, and the mlink procedure of LINKAGE [11] for study NY. We then applied the MAGS method as described above to the linkage results, one chromosome at a time, using a set of analysis points that spanned each chromosome with one analysis point positioned every 2 cM and *D *= 10 cM. We chose *D *= 10 cM because a polymorphism at a marker linked to an analysis point can provide information about the polymorphism at the putative QTL. If an analysis point had only one marker within a 10-cM radius, then no meta-analysis was conducted. For studies DA and KA, we transformed the NPL scores obtained from GENEHUNTER to *p*-values using step 1b; for study NY, we transformed the MaxLOD scores obtained from LINKAGE using step 1a; for study AI, we transformed the *t*-value obtained from SAGE in a similar fashion to the NPL score in step 1b. The subsequent normal variates were weighted by *w*_*stq *_= (1 - 2*θ*_*stq*_)^2 ^*n*_*s*_, where *n*_*s *_is defined as the total number of individuals used in the linkage analysis of study *s*. Note that the weights in this application do not include information content because the measure was not available from all analysis packages (e.g., *Sibpal*). For study AI in which we used *Sibpal*, *n*_*1 *_= 483 possible siblings were included in the analyses. For the studies in which we used GENEHUNTER2 (DA and KA), *n*_*2 *_= 700 and *n*_*3 *_= 694 individuals, respectively, were included in the analyses. For study NY, *n*_*4 *_= 943 individuals were included in the analyses.

We calculated the frequency (across the 100 replicates) that the resulting MAGS values ( for the microsatellite analysis and  for the SNP analysis) exceeded a set critical value at varying alpha levels (*p *= 0.01, 0.001, 7.4 × 10^-4 ^and 2.2 × 10^-5^) [12] to evaluate power and type I error.

## Results

**Figure 1:**
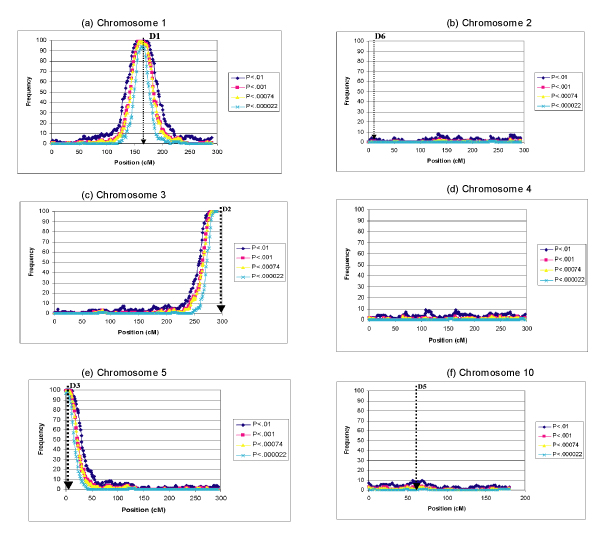
**Frequency (out of 100 replicates) that the MAGS test statistic exceeded the critical value associated with alpha levels of 0.01, 0.001, 7.4 × 10^-4 ^and 2.2 × 10^-5^**  for (a) chromosome 1, (b) chromosome 2, (c) chromosome 3, (d) chromosome 4, (e) chromosome 5, and (f) chromosome 10.

Figure 1 presents the MAGS results for the microsatellite marker maps. The MAGS method identified the disease gene D1 (located approximately at 167 cM on chromosome 1) in more than 90% of the replicates even for very small alpha levels. For disease gene D2 (located approximately at 299 cM on chromosome 3), the MAGS method localized its location in all replicates. Likewise for disease gene D3 (located approximately at 5 cM on chromosome 5), the MAGS method detected its location in more than 90% of the replicates even at alpha levels as low as 2.2 × 10^-5^.

For the disease modifier gene D6 (located approximately at 15 cM on chromosome 2) that affects the penetrance of phenotype 2, the MAGS method was not able to distinguish its signal from that of background genetic noise. Furthermore, D6 was not identified in any of our analyses of the individual studies. Likewise for the modifier gene D5 that is located on chromosome 10 (approximately 67 cM). The location of gene D5 was only identified in 10 of the 100 MAGS replicates when the alpha level was set at 1% and in none of the replicates when the alpha level was set at 2.2 × 10^-5^. Additionally, this gene was not identified in any of our single-study replicates. Meta-analysis of chromosome 4, which did not contain any disease genes, did not detect any erroneous linkage signals for alpha levels less than 1%.

In the analyses using the modified SNP marker maps (data not shown), D1 was identified in over 80% of the replicates while D2 was only identified in 20% of the replicates at an alpha level 2.2 × 10^-5^. Difficulty in identifying D2 was attributable to the sparseness of SNPs in the modified marker maps in the region at the very end of chromosome 3 surrounding D2. As with the microsatellite results, the modifier genes, D5 and D6, were not clearly identified.

## Conclusion

Meta-analysis provides a means of combining information about linkage from smaller independent studies to identify genetic linkage to a complex trait while adjusting for among-study heterogeneity (different sample sizes, different marker maps, etc.). Because multiple genes are believed to be involved in a complex disease, many with modest effects, the probability of identifying them from single studies and replicating the results is low. This meta-analysis procedure correctly identified the three major genes we analyzed with high power even under quite restrictive conditions. Furthermore, this method did not erroneously identify linkage where no linkage was simulated when alpha levels of 0.1% or lower were used. In fact, based on the results for chromosome 4 (where no linkage was simulated) depicted in Figure 1d, the overall chromosome-wide alpha level of 0.1% resulted in point-wise alpha levels of 5% or less. Neither meta-analysis using the SNP or microsatellite data identified the modifier genes directly, but it might be possible to have identified them if the meta-analysis was performed using results from analyses performed conditional on the known genes.

The MAGS method performed better for the microsatellite marker maps than for the modified SNP marker maps. The microsatellite maps had higher marker density than the modified SNP maps with possibly different information content per marker. Also, when we analyzed the modified SNP maps, we did not use the same test for linkage in each study and we did not include information content in the MAGS calculation. The linkage tests that we used (GENEHUNTER2, Sibpal, mlink) vary in the type of pedigree structure and data that is used to test for linkage and hence vary in power to detect linkage which therefore affected the SNP meta-analysis. However, any meta-analytic procedure that is conducted on studies using varying linkage tests (with varying levels power to detect linkage) will be affected by these among-study differences. Meta-analysis provides a way to obtain consensus for linkage to a disease and is clearly an important step in the localization of genes involved in complex diseases.

## Abbreviations

GAW14: Genetic Analysis Workshop 14

KPD: Kofendred Personality Disorder

MAGS: Meta-analysis procedure for genome-wide linkage studies

NPL: Nonparametric linkage

QTL: Quantitative trait locus

SNP: Single-nucleotide polymorphism

GSMA: Genome scan meta-analysis

## Authors' contributions

CJE conceived of the application of this methodology as well as developed the meta-analytic method to the simulated data provided by GAW14. ML conducted the analyses of the simulated data with the assistance of CJE and TJC. All authors contributed to the writing and approval of the final manuscript.

## References

[B1] Wise LH, Lanchbury JS, Lewis CM (1999). Meta-analysis of genome scans. Ann Hum Genet.

[B2] Hedges LV, Olkin I (1985). Statistical Methods for Meta-analysis.

[B3] Rice WR (1990). A consensus combined *p*-value test and the family-wide significance of component tests. Biometrics.

[B4] Province MA (2001). The significance of not finding a gene. Am J Hum Genet.

[B5] Etzel C, Guerra R (2002). Meta-analysis of genetic linkage analysis of quantitative trait loci. Am J Hum Genet.

[B6] Loesgen S, Dempfle A, Golla A, Bickeboller H (2001). Weighting schemes in pooled linkage analysis. Genet Epidemiol.

[B7] Faraway JJ (1993). Distribution of the admixture test for the detection of linkage under heterogeneity. Genet Epidemiol.

[B8] Markianos K, Daly MJ, Kruglak L (2001). Efficient multipoint linkage analysis through reduction of inheritance space. Am J Hum Genet.

[B9] Terwilliger JD, Ott J (1994). Handbook of Human Genetic Linkage.

[B10] Kruglyak L, Daly MJ, Reeve-Daly MP, Lander ES (1996). Parametric and nonparametric linkage analysis: a unified multipoint approach. Am J Hum Genet.

[B11] Lander E, Kruglyak L (1995). Genetic dissection of complex traits: guidelines for interpreting and reporting linkage results. Nat Genet.

[B12] (1997). Department of Epidemiology and Biostatistics, Case Western Reserve University. SAGE: Statistical Analysis for Genetic Epidemiology, Release 31.

